# Case of Dislocation of the Head of the Tibia Forward

**Published:** 1857-03

**Authors:** S. D. Gross

**Affiliations:** Professor of Surgery in the Jefferson Medical College of Philadelphia


ART. VIII.—*Case of Dislocation of the Head of the Tibia forward upon the Thigh-bone.* By S. D. GROSS, M.D., Professor of Surgery in the Jefferson Medical College of Philadelphia.

A VERY large fat woman, weighing nearly two hundred pounds, married, and forty-eight years of age, while engaged in feeding her poultry, sustained a severe fall in consequence of the sudden slip of the right foot, which, bending outwards, thus caused the whole weight of the body to be thrown upon the corresponding knee. I saw her four hours after the occurrence of the accident, when several fruitless attempts had been already made at reduction. The knee, which was very painful and a good deal swollen, especially on the inside, appeared to be unusually wide from side to side; a circumstance partly due to the tumefaction of the soft parts. The leg was one inch and a half shorter than the opposite one, and in a straight line with the thigh. The patella had sunk behind the head of the tibia, into a sort of hollow, which gave to the front of the joint a flattened appearance. Upon grasping the bone, however, with the thumb and fingers, it was easily drawn forwards, leaving a remarkable vacuity behind, in consequence of its distance from the inferior extremity of the femur. The condyles of the thigh-bone lay in the popliteal space, posterior to the head of the tibia, where they formed a large prominence, more distinct on the inside than on the outside, and situated, as it were, in the upper and back part of the leg, the muscles of which were unusally tense. The head of the tibia lay in front of the condyles, where its outlines could be easily traced with the eye and finger. Above this bone, as already stated, was the patella with its ligament and the tendon of the extensor muscles, forming a broad, thick cord in front of the thigh-bone, from which it was removed more than two inches. The leg was easily drawn away from its fellow, but could not be carried inwards, showing that there was extensive rupture of the internal lateral ligament. There was no contusion of the soft parts, nor any discoloration of the integuments.

Chloroform having been administered, a stout lac was applied to the upper part of the thigh, and confided to an assistant, to make the requisite counter-extension, while extension was made by another assistant grasping the foot, the limb being in the extended position. Placing now my left forearm behind the knee, and requesting the aids to pull gently and steadily, I suddenly, with my right hand, bent the leg backwards, and thus in a few seconds effected the reduction; the bone slipping into its proper situation with a distinct "snap." The limb being placed in an easy position, cold cloths were applied to the knee, and a grain of morphia administered to allay pain and prevent spasm.

No untoward symptoms appeared after reduction. The patient kept her bed for nearly a fortnight, and medicated lotions were applied, after the first twenty-four hours, to moderate and subdue inflammation. Purgatives and light diet were also enjoined. In due time passive motion was instituted; the limb was frequently bandaged; and in less than a month from the accident, the woman was able to walk about the house with the aid of crutches. The joint, however, remained weak for a long time, and even now, several years after the occurrence of the injury, the slightest fatigue is attended with temporary lameness.

A complete dislocation of the head of the tibia forward upon the condyles of the thigh-bone is a rare accident, and this is the only instance of the kind that I have ever seen, as an uncomplicated lesion. From the amount of injury sustained by the ligaments of the joint, especially the lateral, severe inflammation may always be expected, which, if not timeously combated, will be likely to eventuate in anchylosis. The limb should, therefore, be carefully bandaged, and maintained perfectly at rest, either upon a bolster, or in the extended position in appropriate splints; free use being made, if necessary, of leeches, and, in all cases, of cold or warm water dressings, simple or medicated, as the circumstances may seem to indicate.

